# Conservation Implications of Changes in Endemic Hawaiian Drosophilidae Diversity across Land Use Gradients

**DOI:** 10.1371/journal.pone.0062464

**Published:** 2013-05-01

**Authors:** Luc Leblanc, Daniel Rubinoff, Mark G. Wright

**Affiliations:** Department of Plant and Environmental Protection Sciences, University of Hawai’i, Honolulu, Hawai’i, United States of America; University of California, Berkeley, United States of America

## Abstract

Endemic Hawaiian Drosophilidae, a radiation of nearly 1000 species including 13 federally listed as endangered, occur mostly in intact native forest, 500–1500 m above sea level. But their persistence in disturbed forest and agricultural areas has not been documented. Thus, control efforts for agricultural pests may impact endemic species if previously undocumented refugia in agricultural areas may play a role in their conservation. To quantify whether invasive plants and agriculture habitats may harbor endemic Drosophilidae, we established standardized trapping arrays, with traps typically designed to control invasive fruit flies (Tephritidae), with 81 sites across native, disturbed and agricultural land use gradients on the islands of Hawai’i and Maui. We collected and identified, to species level, over 22,000 specimens. We found 121 of the possible 292 species expected to occur in the sampled areas, and the majority (91%) of the captured specimens belonged to 24 common species. Species diversity and numbers were greatest in the native forest, but 55% of the species occurred in the invasive strawberry guava belt and plantation forest, adjacent to and almost 500 m from native forest, and 22 species were collected in orchards and nonnative forest as far as 10 km from native habitats. Their persistence outside of native forest suggests that more careful management of disturbed forest and a reassessment of its conservation value are in order. Conservation efforts and assessments of native forest integrity should include the subset of species restricted to intact native forest, since these species are highly localized and particularly sensitive. Additionally, future efforts to control invasive pest fruit flies should consider the nontarget impacts of maintaining traps in and near native forest. This survey project demonstrates the utility of thorough biotic surveys and taxonomic expertise in developing both sensitive species lists and baseline diversity indices for future conservation and monitoring efforts.

## Introduction

Conservation of threatened ecosystems presents a special challenge, distinct from species-centric efforts, since there may be large concentrations of endangered plants and animals vulnerable to extinction in relatively small areas. Further, adequate knowledge of the taxonomy for the more obscure components of biodiversity, like insects, is often lacking, particularly with regard to species-level vulnerability and management needs. Such challenges are global in nature, but perhaps most evident in Hawai’i, which has endured high rates of extinction across a broad range of the flora and fauna. Because most of the macrofauna is extinct [1], [2], insects not only represent the most significant portion of remaining endemicity, but also the best guides to saving overall biodiversity in highly threatened systems. Yet, for the most part, insect biodiversity is poorly known and the varying sensitivity of particular species, even those that are known to be vulnerable, has rarely been studied. A first step towards assessing the conservation needs of an endemic entomofauna, and therefore more broadly conserving their essential roles in native ecosystems [3], [4], should include standardized surveys of insect diversity across gradients representing habitats in various states of degradation. In so doing we could generate data that not only reveals the impacts of land use on a large segment of biodiversity, but also identify those members of the native community which best indicate intact habitat for conservation prioritization. Such a methodology would be of broad utility across many threatened ecosystems, particularly those that have already suffered the extinctions of their most charismatic fauna, but may still harbor important components of the original biodiversity. However, such a methodology requires the use of a group that is adequately diverse so as to provide relative measures of sensitivity to habitat quality across multiple species. It is also necessary to have some level of taxonomic expertise to ensure accurate species level identifications, which dramatically increase the accuracy and value of such surveys.

Endemic Hawaiian Drosophilidae species are an exceptionally diverse assemblage, of approximately 1000 species [5] radiating from a single colonization event [6]. Five hundred and sixty five described endemic species (416 *Drosophila* and 149 *Scaptomyza*) and 32 introduced species are known to occur in Hawai’i [7], [8], [9]. The larvae of the endemic species are extremely specialized, occurring in decaying leaves, bark, fruit, flowers, or the sap flux of plants belonging to 36 angiosperm families, fern rachis, fungus, and even spider eggs or green algae in streams [10], [11], [12], [13]. Most (74.7%) species breed on a single substrate and host plant family and 49% of the Angiosperm-breeding species are monophagous on a single plant genus [13]. Thus, the presence or absence of a suite of drosophilid species may be used to assess habitat integrity and monitor the impact of nonnative plant encroachment across gradients.

The majority of endemic drosophilids occur between 500–1500 m in elevation [14], in four of the Hawai’i forest ecosystems defined by Fosberg [15]: the wet ohi’a (*Metrosideros polymorpha* Gaudich.) forest, the cloud forest (above and contiguous with the ohi’a forest), the drier mixed mesophytic forests, where more unusual hosts including fungi are common, and the dryland sclerophyll forest [16]. Very few species are recorded at lower altitudes, in the leeward dry forest, or the high arid regions above 2100 m [16], and even fewer have ever been collected in nonnative forest [14].

Numerous threats related to human activity and invasive species contributed to the documented decline [17], [18] of endemic drosophilids, to the point that 13 of the picture wings are on the United States Federal Endangered Species list and parts of their critical habitat protected [19]. Major threats include grazing and weed dispersal by feral ungulates, invasions of nonnative plants such as strawberry guava (*Psidium cattleianum* Sabine), conversion of endemic habitats into agricultural and pasture land, wildfires in mesic scrubland, and predation by invasive ants [16], [20] and yellowjackets [18]. Adding to these threats may also be the unintended nontarget impact of practices to control or eradicate insect pests, such as fruit flies (Tephritidae) [21], [22]. In places with very high levels of regional endemicity, like the Hawaiian Islands, such nontarget impacts could threaten species with distributions restricted to a few square kilometers on the slope of a single volcano.

We sought to use the presence of introduced and endemic Drosophilidae to understand the impacts of land use across a gradient on an extremely diverse endemic and introduced insect fauna. We asked how the diversity of both endemic and invasive insects might change through standardized trapping along a transect from endemic forest into adjacent agricultural lands. Specifically, would endemic insects be wholly restricted to intact native forest; which species are more resilient to habitat alteration; is the presence of invasive species indicative of poorer quality habitat; and are any species useful as indicators of not just intact native or agricultural lands, but transitional areas that may also be of some conservation value? To answer these questions we generated data on the occurrence and abundance of 121 endemic drosophilid species across a gradient from intact native forest to intensive agricultural use to help establish a standardized sampling baseline for endemic insect diversity across land use regimes. We present detailed data on the presence of endemic Drosophilidae in native forest, the persistence of populations in adjacent nonnative forest belts, and their presence in more distant agricultural environments on Hawai’i and Maui islands. Because we identified all individual flies to species, specific measures of how much disturbance different endemic species can tolerate is available, as well as the impacts of pest control practices in mixed and native forest. More broadly, this survey data is relevant to the species-specific impact of a land use gradient on a diverse endemic radiation of conservation concern.

## Materials and Methods

### Sites and Traps

**Figure 1:**
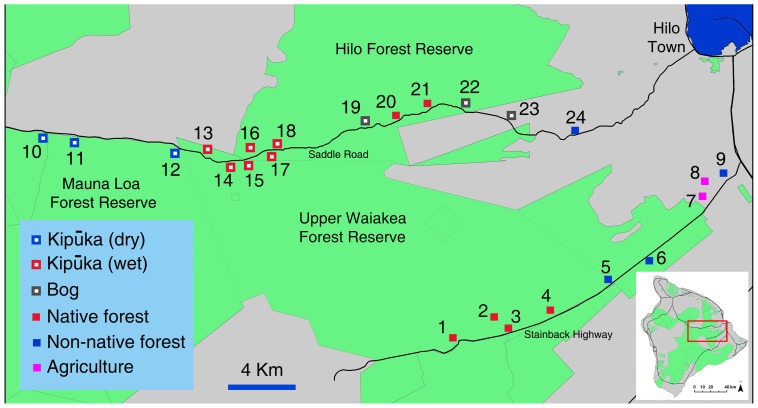
Trapping sites on Hawai’i Island along Stainback Highway and Saddle Road.

**Figure 2:**
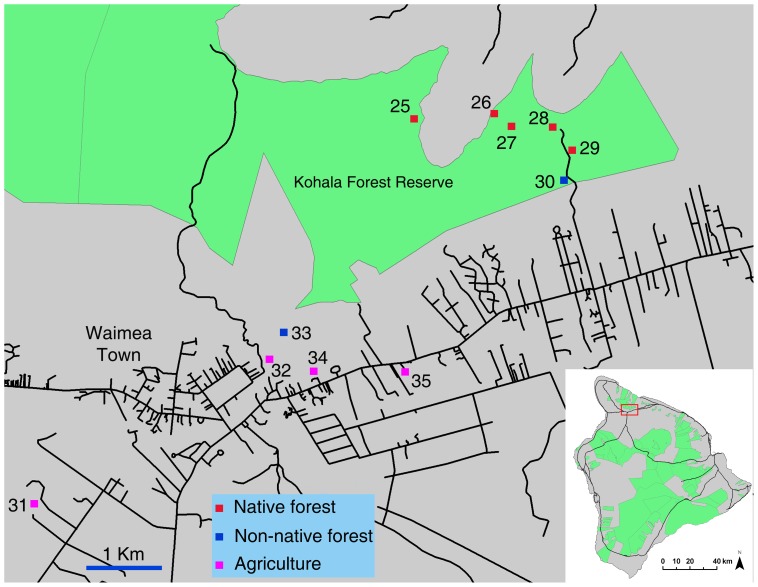
Trapping sites on Hawai’i Island in the Kohala Forest Reserve and Waimea agricultural area.

**Figure 3:**
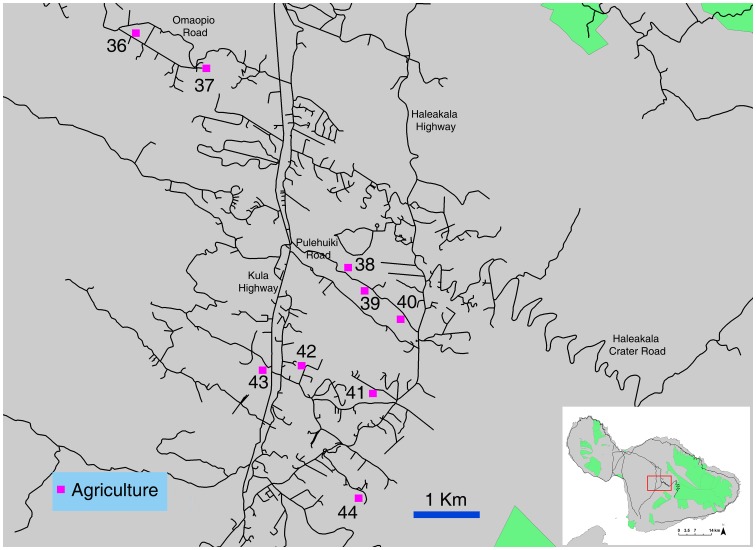
Trapping sites on Maui Island in the Kula agricultural area.

**Figure 4:**
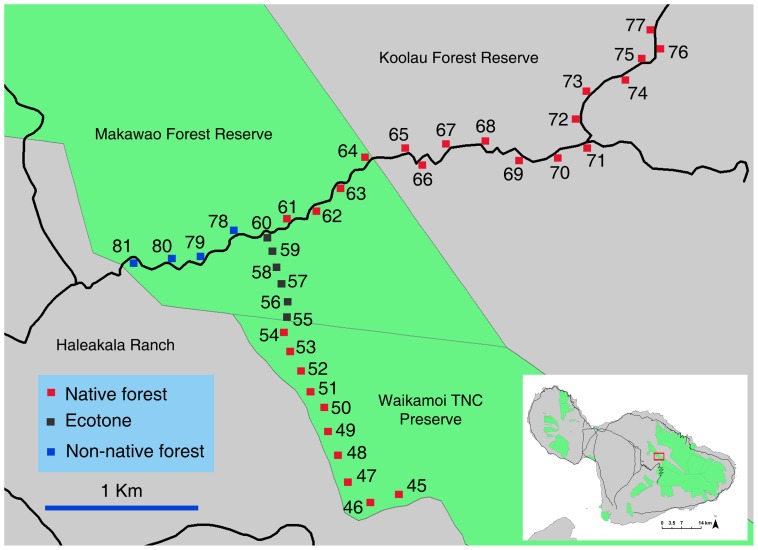
Trapping sites in the forest reserves on Maui Island.

We surveyed endemic and introduced drosophilids using a standard trapping protocol established to monitor nontarget insect attraction fruit fly male lures and food attractants [21], [22]. Traps were maintained at 81 sites, in six broad locations on Hawai’i and Maui Islands, covering a diversity of ecosystems, from farmland to invasive forest, strawberry guava belts, mixed and endemic forest. While trapping procedures across sites were not always precisely replicated, they were consistent within each gradient, allowing for comparison across land use regimes. All sites, referred to by their numbers throughout this paper, are described in details and mapped on Figure 1, Figure2, Figure 3, and Figure 4.

### Ethics

Permits for collecting insects in the State Forest Reserves were delivered by the Hawai’i State Department of Land and Natural Resources (Betsy Gagné) and access permits were granted by its Division of Forestry and Wildlife offices. Required additional access permits to the Waikamoi Preserve and the Ko’olau Forest Reserve were granted by the Nature Conservancy (Pat Bily) and the East Maui Irrigation Company, respectively. We also acknowledge the private landowners and growers in Waimea and Kula for their hospitality and permission for access and use of their farms.

### Hawai’i Island

Nine sites were maintained in a 20 km long transect along the Stainback Highway, from the Pana’ewa Rainforest Zoo near Hilo (138 m above sea level) up to 1,045 m. The upper four sites (1,045–706 m) (sites 1 to 4) were in native wet montane ohi’a-dominated forest, and the lower sites 5 to 9 (522–138 m) were in invasive strawberry guava dominated forest (three sites), a citrus orchard and a mixed fruit orchard. Fifteen additional sites were in a 35 km transect on the Saddle Road, from the junction of Kaūmana and Saddle Roads (439 m) to Pu’u Huluhulu (2,012 m) (sites 10 to 24). The lowest site was in invasive strawberry guava forest, while the other sites were in native montane wet herbland bogs over recent lava flows (three sites), wet montane ohi’a forest (two sites), and wet (six sites) and dry (three sites) ohi’a-dominated kīpuka forests. Six sites were along the upper Hāmākua Ditch Trail (North Kohala Forest Reserve), from the far end of the flume (1,019 m) to the entrance of the Reserve, off the Waimea water reservoir (906 m) (sites 25 to 30). The forest entrance site was in the strawberry guava belt, adjacent to the mixed native wet montane ohi’a forest (where the other sites were located). Five trapping sites were in the agricultural community of Waimea (744–872 m), about 4 km southwest of the Kohala sites (sites 31 to 35). Two sites were in backyards with a diversity of fruit trees, one site in a citrus orchard, one site in a large feral stand of common guava (*Psidium guajava* L.), and the last site at the foot of the North Kohala Forest Range, in a forest dominated by invasive tropical ash [*Fraxinus uhdei* (Wenzig) Lingelsh].

### Maui Island

Fourteen sets of traps were maintained in nine sites across the agricultural community of Kula (517–1,138 m) (sites 36 to 44). Sites covered a variety of common tree crops: persimmon (*Diospyros kaki* L. fil.) orchards (six sets of traps), coffee plantations (two sets in maintained and two sets in abandoned plots), two sets in nonnative forest adjacent to orchards (one next to persimmon and the other next to persimmon and coffee), and two sets in citrus and mixed fruit orchards. Forest ecosystems on Maui were covered with a 37-site transect, on the northern slope of the Haleakalā Volcano, with one site every 150 m, along two linear intersecting transects (sites 45 to 81). The first 2 km long transect was along the Maile Trail, from the Flume road in the Makawao Forest Reserve (1,294 m) (mixed native/invasive forest) into the Waikamoi Nature Conservancy Reserve (intact mesic forest dominated by ohi’a and koa) up to near ‘Ukulele Camp at 1,583 m (sites 60 to 45). The second (4 km) transect was along the Flume Road, from the entrance of Makawao Forest Reserve (1,284 m) first along nonnative plantation forest dominated by *Pinus* sp., *Eucalyptus* sp., or tropical ash (sites 81 to 78), then into mixed native mesic forest (sites 60 to 64), continuing into the Ko’olau Forest Reserve (wet native ohi’a-koa forest) to the junction of the Pipeline Road (1,285 m) (sites 65 to 71), and for 1 km along the Pipeline Road forest down to 1,184 m elevation (mixed native/invasive wet forest) (sites 72 to 77).

### Traps and Lures

We surveyed using the bucket and MultiLure traps typically used for fruit fly monitoring, since these not only attracted endemic insects but also allowed us to evaluate the potential for nontarget impacts of pest fruit fly suppression. Additionally, at some sites, we used mushroom-bait and pan traps, which are known to be attractive to endemic insects [23], [24].

Bucket traps [21] consisted of 1.3-liter white plastic cups with two lateral holes on opposite sides near the top to allow insect entry; they were covered with a plastic plate to prevent trap flooding by frequent rain. Aluminum tie wire was used to hang traps in trees. Each trap had one of four lure treatments. For male lure treatments, lure plugs with the fruit fly attractants cue-lure or methyl eugenol (Scentry Biologicals, Billings, Montana, USA) were suspended from the trap inner hook. For the third treatment, decaying fruit flies [*Bactrocera dorsalis* (Hendel)] were placed in pouches made of gauze at the bottom of the trap, in the liquid preservative, to emulate the accumulation of decaying fruit flies attracted to male lures, since many insects are attracted to this resource [21]. The last treatment was an unbaited control trap. Bucket traps baited with fermented mushroom bait in a saturated 4×2×2 cm sponge hung below the trap ceiling [23] were used, instead of traps baited with decaying flies, at the Maui forest sites. A 25×45 mm strip containing 10% dichlorvos (Vaportape ® II, Hercon Environmental, Emingsville, Pennsylvania, USA) was attached to the inner hook of all traps, to rapidly kill captured arthropods.

To more completely assess the drosophilid diversity across sites, we also used MultiLure® traps (Better World Manufacturing Inc, Fresno, CA), baited with the fruit fly food attractant BioLure® (Suterra LLC, Bend, OR), consisting of three components (ammonium acetate, trimethylamine hydrochloride, and putrescine) in separate packets with slow-release membranes, attached to the inner surface of the trap cover.

Yellow pan traps, made of yellow Solo® 12oz plastic bowls (15 cm diameter, 4 cm deep) (Solo Cup Company, Lake Forest, Illinois, USA) were used in the Maui forest sites. They effectively attract a broad range of flying insects, including Drosophilidae [24].

Additionally, 200 ml of a 20% solution of propylene glycol (Sierra Antifreeze®, Old World Industries, Northbrook, Illinois, USA) was used in all the traps to retard decay of captured arthropods, and facilitate identification of specimens.

### Trapping Procedure

At each site, traps were hung in trees 1.5–2.0 m above the ground, and at least 10 m apart to avoid interference between traps. Trap contents were cleared weekly, unless otherwise indicated. Traps on Hawai’i Island, four buckets and one BioLure per site, were maintained from June to August 2005, for 10 weeks on Stainback, 9 weeks on Saddle and in Waimea, and 8 weeks in Kohala. Bucket traps with the male lure traps and the lure-free controls were maintained and serviced continuously through the season. Traps with decaying flies as bait and the BioLure traps were maintained continuously at Waimea sites and for one week straight, every other week, in Stainback (five collections starting at week 1) and Kohala and Saddle (three collections starting at week 3). In Kula (Maui), four buckets and one BioLure trap were set at each site starting in May 2006, near the end of the persimmon flowering season, and left until the end of harvest season, in late November. Traps were serviced weekly for 13 weeks (until late August), and subsequently monthly for the last 3 months. Traps baited with decaying flies were used only during the first 13 weeks, because monthly servicing intervals would have caused a complete decay of the trapped drosophilids themselves. In the Maui forest transects, we maintained at each site three bucket traps (both male lures and the unbaited control) continuously for 12 weeks (June-August 2006). After 6 weeks, all trap sites were shifted downslope by 75 m along the transects, to maximize habitat coverage. To avoid impacting endemic insect populations, BioLure traps were intermittently used for 14 days, as 3 days on week 3, 4 days on week 4, and again 3 days on week 9 and 4 days on week 10. Traps with decaying flies were not used in the Maui forest, because similar or closely related species present there were previously found to be attracted to BioLure on Hawai’i Island [21] and we did not want to overly impact populations of particular species by trapping them more intensively. Instead, we maintained mushroom baited traps, one at each site, intermittently for one week on weeks 4, 6, 10 and 12, as well as pan traps, three at each site, placed on the ground and maintained for 7 days, on weeks 4 and 10 to broadly assess drosophilid diversity.

Positions of traps at all sites on both islands were re-randomized every 3 weeks to minimize effect of trap position on catches. Pouches of decaying flies were replaced weekly, while male lure plugs, pesticide strips and BioLure membranes were used for the duration on Hawai’i Island and Maui forests, and replaced once after 13 weeks in Kula.

### Sample Processing and Data Analysis

All Drosophilidae were counted, sexed and identified to species level whenever feasible. Reference collections of voucher specimens have been deposited at the University of Hawai’i Insect Museum (Mānoa), and the Bishop Museum, in Honolulu (HI).

Trapping data for endemic Drosophilidae are reported here, with the alien species reported in more details separately [7]. All counts from each sample were converted to number of flies per trap per day. Because drosophilids were not attracted to the male lures [21], capture data from bucket traps with the two male lures and the unbaited control traps were analyzed together, as bucket traps. Similarly, traps with decaying flies, BioLure and mushroom bait attracted comparatively large numbers of mostly the same species of drosophilids, and their data are treated together under the “food lure” category. Data from pan traps are treated separately. The EstimateS software [25] was used to generate the species accumulation curve, with 50 randomizations without replacement.

To analyze differences in fly community assemblages in each of the habitats sampled, mean number of flies collected in traps was calculated across sampling dates within each trapping site. Canonical correspondence analysis (CCA) was used to analyze fly community structure at the “species group” level in the different habitats, and to describe effects of environmental factors on community composition and distribution of fly species in different habitats, using CANOCO 4.5 [26]. Problems typically associated with unbalanced experiments are reduced by screening for collinearity among environmental variables (habitats) using the inflation factor in CANOCO, which reduces potential bias [26], [27]. Environmental variables included in the analysis were habitat type (endemic forest, kīpuka and bogs, mixed endemic/non-indigenous forest, nonendemic forest, fruit and coffee orchards, and invasive strawberry guava or common guava). Fly counts were log n+1 transformed, and rare species were downweighted in the analyses [26]. Significance of correspondence axes was assessed using Monte Carlo tests (499 permutations), as implemented in CANOCO 4.5. These analyses were conducted on species groups of Drosophilidae [8], and on individual species in separate analyses. For the species group analysis, data from both islands were combined, as the members of the groups share similar ecological characteristics [13], and members of all groups were present on both islands. The species level analyses were separated by island, because very few species were collected on both islands. Preliminary examination of the data indicated that a linear model was more appropriate than a unimodal model, and therefore Redundancy Analysis (RDA) was used to analyze the species occurrence in different habitats (CANONO 4.5). Results of the ordinations were plotted (CCA or RDA axes 1 and 2) with environmental variables to visualize the changes in community assemblages in different habitats, and associations of species groups and species with specific habitats.

## Results

**Figure 5a–d:**
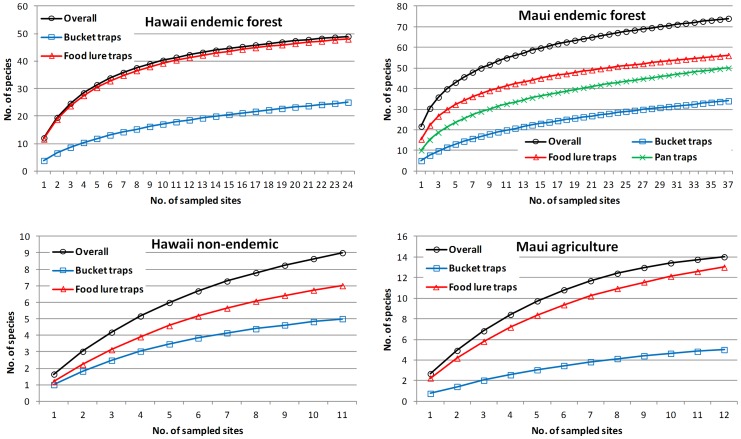
Species accumulation curves for endemic Drosophilidae collected in Hawai’i. Data presented separately for different types of traps in endemic forest sites of Hawai’i (a) and Maui (b) and nonnative forest and agricultural sites of Hawai’i (c) and Maui (d).

Two hundred and ninety-one of the 328 endemic Drosophilidae species from Maui and Hawai’i Islands were known or likely to occur in the sampled areas, on the basis of literature and museum specimen label data. We collected over 22,000 specimens, representing 121 of these expected species, plus 32 undescribed new species, suggesting our sampling methods were relatively thorough (Table 1). Species richness was higher on Maui (75 species) than Hawai’i (50 species). Additional sampling would have revealed additional species, as reflected in the species accumulation curves (Fig. 5), but locating nearly half of the known species in the study sites, and such large numbers, allows for inference regarding the distribution of these endemic insects with respect to habitat use. The contribution of different trapping methods to detecting a more saturated sample of species richness in the different habitats is demonstrated in Fig. 5; if only a single method had been used, the species accumulation curves would have undersampled the drosophilid assemblage substantially. While food lures attracted large numbers of the spoon tarsus, *Antopocerus*, *Engiscaptomyza*, *haleakalae* and picture wing groups, bucket and pan traps collected a number of other species underrepresented in food lure samples, such as the *Elmomyza* on Hawai’i island and the two most numerous of the modified mouthpart species in our samples, *D. comatifemora* and *D. hirtitarsus*, caught almost exclusively in the pan and bucket traps, respectively. Similarly, the two bristle tarsus species were collected exclusively in pan traps on Maui. Other species may turn out to be widespread when non-traditional sampling methods are used, as for *S. undulata*, thought to be very rare until pan traps were found to collect them in large numbers [14]. The complete list of the species collected, their island-wide distributions, on the basis of our data, literature surveys and museum data, is presented in Table S1 (supporting information) to this paper and as part of a searchable database (Drosophilidae of Hawai’i. Available: www.herbarium.hawaii.edu/drosophila/Accessed 2013 Mar 8).

**Table 1:** Numbers of expected and captured species of Drosophilidae during studies of nontarget attraction to fruit fly (Tephritidae) lures on Hawai’i and Maui Islands, summarized by group.

		Number of species			Proportion of total endemic species captured		
Group	Total described in Hawai’i	Expected at sites	Collected at sites	New species	Hawai’i Is.	Maui	Hawai’i and Maui
Endemic *Drosophila*							
* Antopocerus*	15	8	7	0	35.46	5.69	16.33
* Bristle tarsus*	18	14	4	6	0.06	0.13	0.10
* Ciliate tarsus*	21	10	4	4	0.56	0.37	0.44
* haleakalae*	54	29	21	1	6.11	31.26	22.28
* Modified mouthparts*	106	54	7	5	5.42	7.39	6.69
* Nudidrosophila*	28	7	2	0	0.42	0.63	0.55
* Picture wing*	120	50	20	0	1.74	2.65	2.32
* Split tarsus*	24	15	8	1	5.18	3.77	4.27
* Spoon tarsus*	12	10	9	0	19.02	9.65	13.00
Misc and unplaced	18	6	1	0	0.00	0.05	0.03
Endemic *Scaptomyza*							
* Alloscaptomyza*	8	4	2	3	0.46	0.26	0.34
* Bunostoma*	8	5	4	0	0.01	0.50	0.32
* Celidosoma*	1	0	0	0	0.00	0.00	0.00
* Elmomyza*	86	55	22	4	22.81	24.72	24.04
* Engiscaptomyza*	6	4	2	0	0.00	12.04	7.74
* Exalloscaptomyza*	6	2	1	0	0.00	0.02	0.01
* Grimshawomyia*	3	3	1	0	1.25	0.00	0.44
* Rosenwaldia*	6	3	0	0	0.00	0.00	0.00
* Tantalia*	6	3	2	1	0.00	0.72	0.46
* Titanochaeta*	12	7	3	7	1.22	0.15	0.53
Unplaced *Scaptomyza*	7	3	1	0	0.27	0.00	0.10
Immigrant Drosophilidae	32	29	23	0	n.a.	n.a.	n.a.

**Figure 6:**
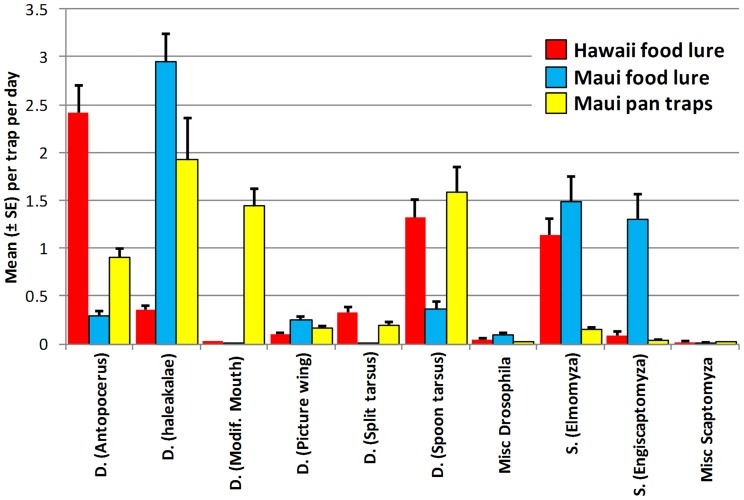
Mean (± SE) captures of endemic *Drosophila* and *Scaptomyza*, summarized by taxonomic groupings. Data presented based on captures in food lure and yellow pan traps maintained in native forest sites on Hawai’i (2005) and Maui (2006) islands.

The dominant groups, in number of specimens collected and the highest proportion of expected species actually captured, were the leaf breeder (*Antopocerus*, spoon tarsus, split tarsus) and fungus breeder (*haleakalae* group) groups of *Drosophila*, and the subgenera *Elmomyza* and *Engiscaptomyza* of *Scaptomyza* (Table 1, Fig. 6). *Antopocerus*, spoon tarsus, and *Elmomyza* accounted for 77% of all captures on Hawai’i Island, and *haleakalae*, spoon tarsus, *Elmomyza* and *Engiscaptomyza* were numerically dominant on Maui, with 78% of all captures. The majority (91%) of captures were of 24 common species (online support material). New island distribution records are also reported for fourteen species, further demonstrating the effectiveness of our survey methods: *D. brunneisetae* Hardy, *D. macrochaetae* Hardy, *S. chauliodon* (Hardy), *S. cryptoloba* Hardy, *S. mutica* Hardy are new to Hawai’i Island, and *D. paucitarsus* Hardy & Kaneshiro, *S. articulata* Hardy, *S. basiloba* Hardy, *S. brunnimaculata* Hardy, *S. diaphorocerca* Hardy, *S. levata* Hardy, *S. nigrosignata* Hardy, *S. setosiscutellum* (Hardy), and *S. xanthopleura* Hardy are new to Maui.

Host plant and breeding substrate are known for 71 of the 121 collected species, and most breed in the leaves and/or bark of either the Araliaceae (*Cheirodendron* and *Tetraplasandra* spp) (34 species) and/or the Campanulaceae (mostly *Clermontia* spp) (19 species), or on fungi (12 species), suggesting strong associations with the native flora.

Only four species of the charismatic picture wings were found on Hawai’i, all infrequently (except *D. ochracea* Grimshaw), consistent with their documented decline in the island’s wet forests attributed to the destruction of *Clermontia* host trees by feral pigs and predation by invasive yellowjacket wasps [17], [18], [28]. In contrast, 14 species of picture wings were regularly trapped in the Maui native forest, especially in the Waikamoi reserve, where host *Clermontia* trees abound and efforts are underway to eradicate pigs from the fenced reserve.

At least a few individuals of 23 of the 29 expected introduced drosophilid species were collected [7], but three species dominated the captures and were common even in the endemic habitats (Table 2): *D. sulfurigaster bilimbata* Sturtevant (47% of introduced drosophilids), *D. immigrans* Bezzi (4%) and *D. suzukii* (Matsumura) (46%). The first two breed on decaying guava [14] and other introduced fruit, rather than the usual substrates of the endemic species, and *D. suzukii*, a severe pest of small fruits abundant at all trapping sites, was bred from endemic raspberry (*Rubus hawaiiensis* A. Gray) [13]. Assuming that *D. suzukii* can infest most or all of the nine species of *Rubus* of Hawai’i, and possibly the endemic ōhelo (*Vaccinium* spp) as well, then hosts are commonly available from sea level to at least 2000 m, in mesic to wet environments [29], potentially sustaining the large populations of this pest fly observed in Hawai’i. Its impact as a potential pest of endemic raspberry has not been documented. Since guava is invasive and not used as a host by endemic drosophilids, the potential impact of the most common invasive drosophilids in the Hawaiian forest is likely to be limited. Possible competition of *D. immigrans* with endemic species for breeding sites has been suggested [14], but not yet investigated.

**Table 2:** Mean captures (mean ± SE per trap per day) of endemic and immigrant Drosophilidae in food lure and bucket traps in endemic and adjacent ecotone or nonendemic habitats.

Transect	Habitat	No.trapping sites	No. endemic species trapped	Endemic drosophilids in food lure traps	Endemic drosophilids in bucket traps	Immigrantdrosophilids infood lure traps	Immigrant drosophilids in bucket traps
Stainback	Endemic	4	22	4.09±0.76	0.27±0.05	13.29±5.63	0.06±0.02
	Nonendemic	5	5	0.04±0.01	0.03±0.01	247.9±74.0	7.41±2.44
Kohala	Endemic	5	18	10.68±1.51	0.23±0.03	12.73±2.23	0.12±0.02
	Ecotone	1	10	4.89±0.80	0.24±0.06	35.8±24.4	0±0
Waimea	Nonendemic	5	5	0.19±0.05	0.02±0.01	11.18±1.79	1.90±0.56
Saddle	Endemic	14	42	5.13±0.71	0.27±0.02	3.64±0.52	0.03±0.01
	Nonendemic	1	1	0.02±0.02	0.02±0.01	206±185	1.50±0.66
Maui forest	Endemic	28	70	8.17±0.73	0.28±0.02	4.99±0.70	0.01±0.001
	Ecotone	5	33	3.04±0.95	0.05±0.01	5.56±1.60	0.004±0.002
	Nonendemic	4	24	2.25±0.56	0.07±0.02	9.93±2.00	0.002±0.001
Kula	Nonendemic	9	14	0.05±0.01	0.01±0.002	2.34±0.41	0.08±0.01

**Figure 7:**
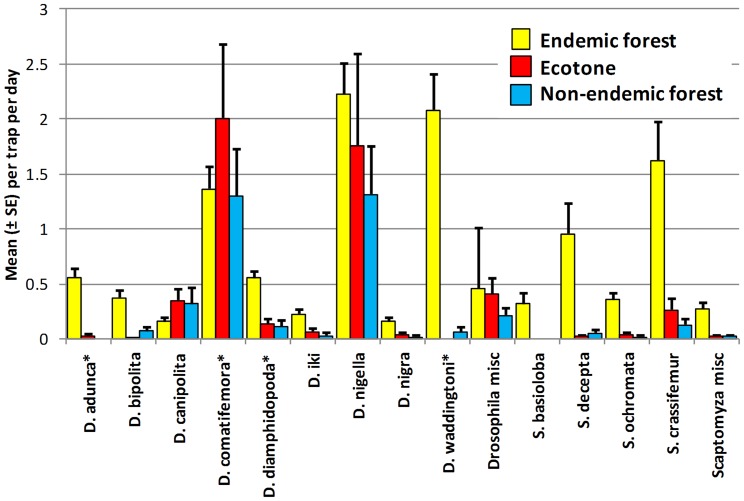
Mean (± SE) captures of endemic *Drosophila* and *Scaptomyza* in native forest and adjacent strawberry guava belt. Sites located in the Kohala Forest Reserve, Hawai’i Island (2005).

**Figure 8:**
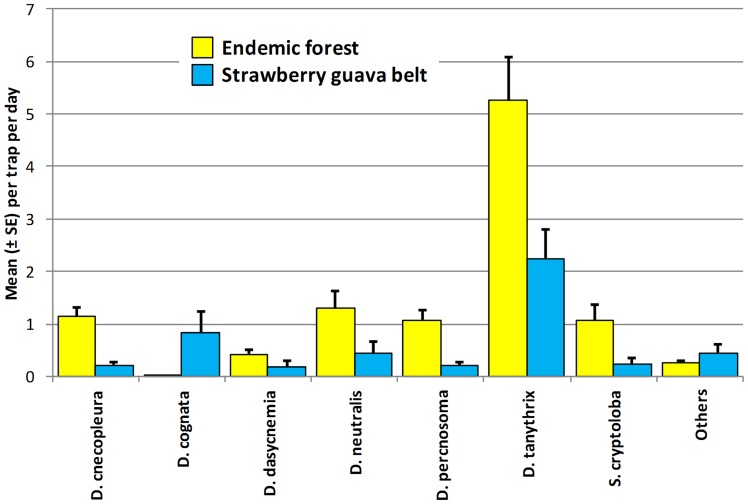
Mean (± SE) captures of endemic *Drosophila* and *Scaptomyza* in native and adjacent ecotone and nonnative forest. Sites located in the Makawao-Waikamoi-Ko’olau forest transect of Maui Island (2006), in endemic forest, nonendemic ecotone within endemic forest, and nonendemic forest adjacent (up to 400 m distant) to endemic forest. Data are from captures in food lure traps (mushroom bait and BioLure), except for species with asterisks (*), for which pan trap capture data are used, because of their higher captures than in food lure traps.

Endemic species were also captured in nonnative forest, adjacent to endemic habitats (Table 2). In the Kohala Forest Reserve on Hawai’i, they were common in the strawberry guava forest belt (site 30), almost 100 m from endemic forest (Fig. 7). In the Maui forest transect, six of the 13 most common endemic species were regularly captured in mostly nonnative forest patches in the endemic forest reserves (sites 55–60), and in adjacent nonnative plantation forest (sites 78–81), up to almost 500 m distant from endemic forest (Fig. 8). These distant occurrences suggest that either they are breeding in the nonnative habitat or they are dispersing through it. In either case it has implications for nontarget impacts of pest tephritid fruit fly control. Similarly, three endemic species were trapped in alien mountain ash forest, almost 500 m distant from endemic forest in the Kohala Mountains (site 33). One of them, *S. lobifera* Hardy, occurred beyond the forest in feral guava stands (site 32) and a backyard (site 35), at least 1 Km from the native Forest Reserve.

Although the majority of endemic flies were collected in endemic forest sites and adjacent nonnative forest, a diversity of endemic species were captured in small numbers in farmland and nonnative forest and orchards, more than 10 Km from endemic forest (Table 3, Fig. 5c–d). On Maui, fourteen species were repeatedly trapped at four of the Kula farmland locations, in coffee and persimmon orchards and adjacent nonnative forest, more than 5 Km from any endemic forest. Larvae of several of these taxa breed in fungus, and may not be host specific, possibly rendering them less effective for habitat quality assessments, and simultaneously more vulnerable to nontarget impacts of fruit fly control. Endemic drosophilids in Kula were more common in the nonnative forest adjacent to orchards (0.11 per trap per day) than in the orchards themselves (0.004 per trap per day) [22]. Because pest fruit flies were uncommon in these forest patches, growers can avoid impacting fringe populations of endemic insects by restricting trapping and protein bait spraying to the orchards themselves [22]. Similarly, six endemic species were trapped at all five lower sites of the Stainback Highway, even in strawberry guava forest and fruit orchards. The picture wing *D. ochracea*, which breeds on bark of *Freycinetia arborea* Gaudich, was repeatedly trapped in four of the five Stainback sites, despite being a relatively uncommon species in Ola’a [18]. This suggests that sparsely distributed *Cheirodendron* and other host trees observed at low altitude along Stainback Highway may sustain small populations of endemic drosophilids in otherwise inhospitable disturbed environments. Such information is of great relevance for conservation planning and habitat restoration.

**Table 3:** Captures of endemic *Drosophila* and *Scaptomyza* in bucket and food lure traps maintained in nonendemic forest and agricultural ecosystems on Maui and Hawai’i Islands.

Location/species	Total captured	No. sites	Habitats	Known hosts^a^
Maui: Kula				
* D. fuscifrons*	10	2	Persimmon, forest next to orchard	Unknown (possibly fungus)
* D. hirtitarsus* b	118	1	Coffee, persimmon, forest next toorchard (mostly)	Sap flux of *Nestegis* (Oleaceae)
* D. polita*	23	2	Persimmon, forest next to orchard(mostly)	Fungal body
* D. quinqueramosa*	2	2	Forest next to orchard	Unknown (possibly fungus)
* S. buccata*	3	1	Persimmon	Unknown
* S. confusa*	47	3	Coffee (mostly), persimmon	Unknown
* S. crassifemur*	1	1	Coffee	Unknown
* S. decepta*	15	2	Coffee, persimmon, forest next toorchard	Unknown
* S. diaphorocerca*	2	2	Coffee, forest next to orchard	Unknown
* S. hackmani*	1	1	Forest next to orchard	Leaf, flower, fruit or bark of *Cheirodendron* (Araliaceae), *Clermontia* (Campanulaceae), *Rubus* (Rosaceae) and *Melicope* (Rutaceae)
* S. levata*	1	1	Persimmon	Unknown
* S. mauiensis*	2	2	Persimmon, coffee	Flowers of *Ipomoea* (Convolvulaceae)
* S. nasalis*	3	1	Coffee, forest next to orchard	Unknown
* S. xanthopleura*	21	3	Coffee, persimmon, forest next toorchard	Fungal body
Hawai’i: Waimea				
* D. bipolita*	5	1	*Fraxinus uhdei* forest	Unknown (possibly fungus)
* D. cnecopleura*	1	1	Backyard	Leaves of *Cheirodendron* (Araliaceae)
* S. chauliodon*	1	1	*Fraxinus uhdei* forest	Spider egg mass
* S. lobifera*	57	3	*Fraxinus uhdei* forest, commonguava stand, backyard	Unknown
* S. palmae*	1	1	Backyard	Palm, flowers of *Hibiscadelphus* (Malvaceae)
Hawai’i: Stainback				
* D. bipolita*	3	2	Strawberry guava, invasive forest	Unknown (possibly fungus)
* D. hirtitarsus* (sp nr)^b^	19	2	Strawberry guava, invasive forest	Unknown
* D. infuscata*	2	2	Strawberry guava, invasive forest	Stem and bark of *Clermontia* (Campanulaceae), *Nestegis* (Oleaceae), and *Freycinetia* (Pandanaceae)
* D. murphyi*	1	1	Mixed fruit orchard	Bark of *Cheirodendron*, *Tetraplasandra* (Araliaceae) and *Clermontia* (Campanulaceae)
* D. ochracea*	19	4	Strawberry guava, invasive forest, fruit orchards	Stem and bark of *Freycinetia* (Pandanaceae)
* S. cryptoloba*	4	2	Strawberry guava, invasive forest	Leaf/frass of *Charpentiera* (Amaranthaceae) and *Clermontia* (Campanulaceae)

a Host record information from reference 13.

b *D. hirtitarsus* is complex of very similar species, with the true *D. hirtitarsus* restricted to Maui, and those on the others islands are yet unstudied sibling species (35).

**Figure 9:**
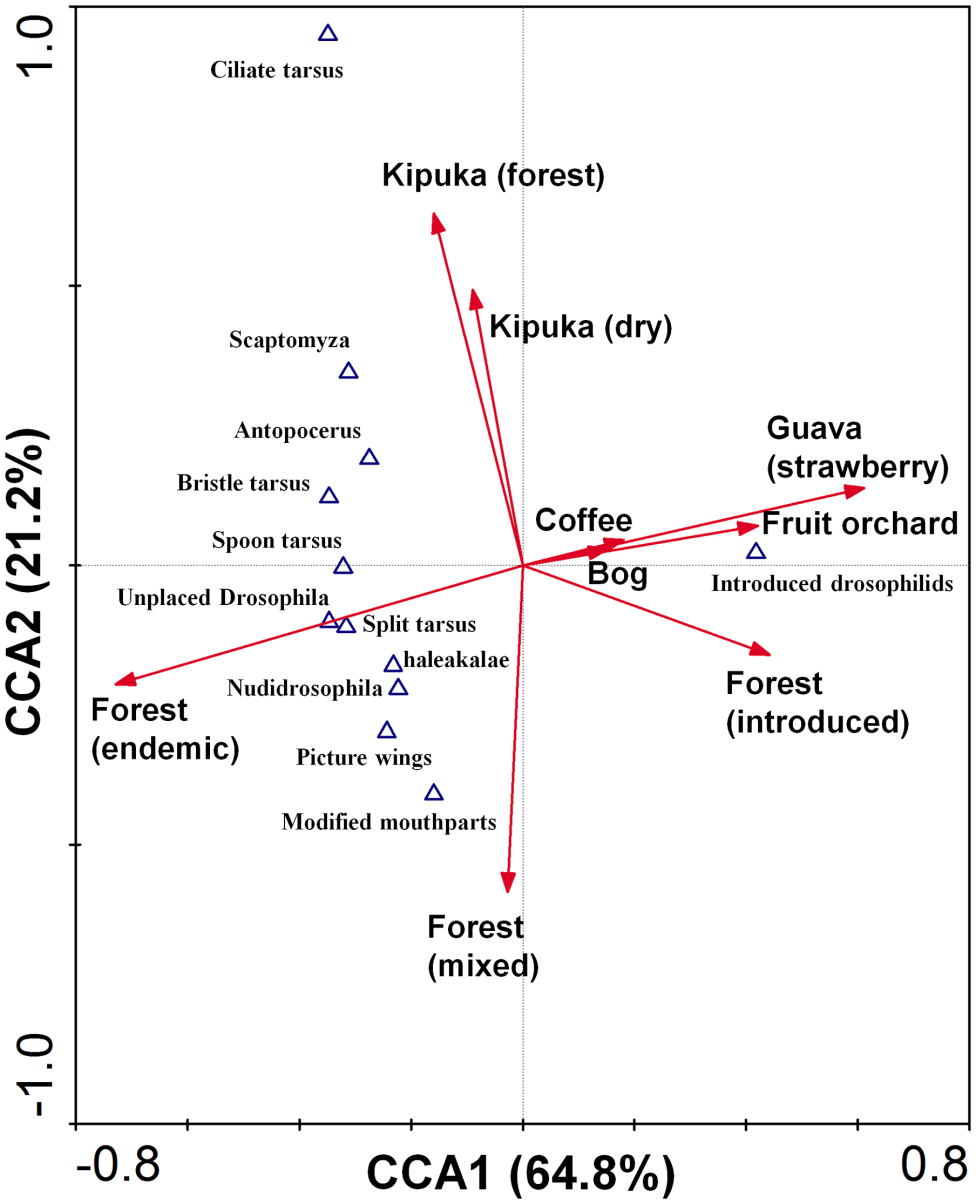
Canonical correspondence analysis biplot of Drosophilidae groups and habitat types sampled in Hawai’i. The length of arrows shows the degree of influence of each habitat variable.

Canonical correspondence analysis (CCA) of the species groups data (Fig. 9) showed a distinct gradient from endemic to invasive plant habitats (CCA axis 1, *P* = 0.0020; species-environment relation variance explained by axis 1∶64.8%; and cumulatively by axis 1 and 2∶86.0%), with immigrant species consistently associated with strawberry guava and fruit orchards, and to a lesser extent, introduced forest species and coffee or bog habitats. While some endemic groups (e.g. modified mouthparts) were associated with mixed forest and primarily endemic forest, the majority of endemic groups were most closely associated with endemic forest, and were only captured in low numbers in mixed and introduced forest habitats. The second axis on the CCA ordination for species groups was defined primarily by mixed forest, and kīpuka habitats (Fig. 9). Some groups (notably the ciliated tarsus clade), were closely associated with the Saddle Road Kīpuka habitats, and were completely unassociated with alien habitats.

**Figure 10:**
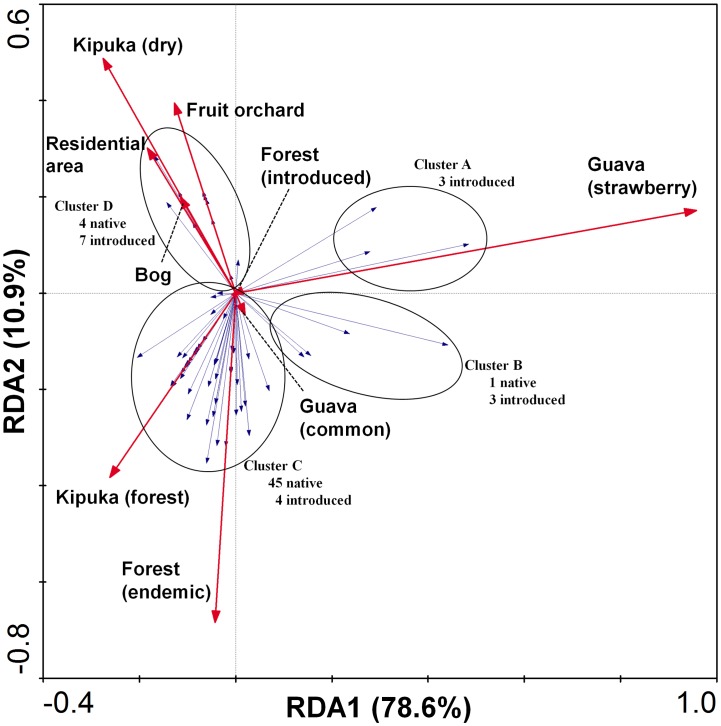
Redundancy analysis biplot of Drosophilidae species and habitat associations on Hawai’i Island. Species included in each group are listed in Table S1 (supporting information). Length of habitat vectors indicates influence of each on the ordination; species vectors indicate strength of association of each species with any habitat vector; cosine of the angle between vectors estimates correlation, smaller angles show higher correlations.

Redundancy Analysis (RDA) results for individual species on each island are presented with the fly species grouped into clusters, circling delineated by eye, on the RDA ordinations for clarity of presentation. RDA on Hawai’i Island (Fig. 10) showed a marginally significant (*F* = 12.46; *P* = 0.0640, species-environment relation variance explained by axis 1∶78.6%; and cumulatively by axis 1 and 2∶89.5%) association of adventive species with strawberry guava (clusters A and B, right quadrants of RDA ordination). A further distinct group of species (cluster C, lower left quadrant, dominated by endemic species), was associated with Kīpuka habitat and endemic forest, with a minimal association of those species with introduced forest and common guava. The relatively wide spread in the ordination of the 45 species in cluster C along the second RDA axis indicates that this assemblage of species is associated with endemic forest, but do occur to some extent in adjacent introduced forest. A smaller set of four endemic species and seven introduced species (cluster D, upper left quadrant), were associated with fruit, bog and residential area habitats.

**Figure 11:**
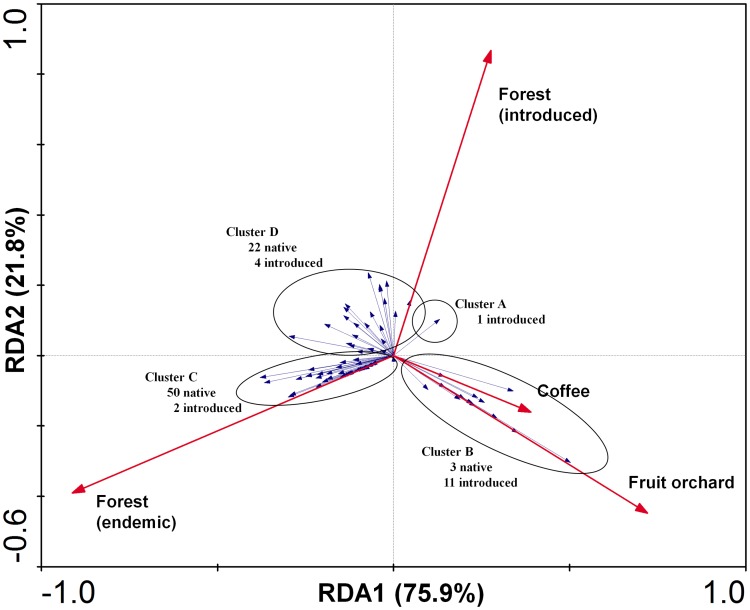
Redundancy analysis biplot of Drosophilidae species and habitat associations on Maui. Species included in each group are listed Table S1 (supporting information). Length of habitat vectors indicates influence of each on the ordination; species vectors indicate strength of association of each species with any habitat vector; cosine of the angle between vectors estimates correlation, smaller angles show higher correlations.

RDA for the Maui (Fig. 11) samples showed highly significant (*F* = 7.64; *P* = 0.0080; species-environment relation variance explained by axis 1∶75.9%; and cumulatively by axis 1 and 2∶97.7%) clusters of species associated with fruit and coffee plantations (lower right quadrant), an intermediate group associated, to some extent, with most habitats examined (clustered in upper left and right quadrants), albeit most strongly associated with endemic- and introduced forest habitats, and a third cluster associated primarily with endemic forest. Clusters A and B are distinctly separated from clusters C and D in the diagram. Clusters C and D are essentially not separable along the first RDA axis (Fig. 8), but the second RDA axis appears to distinguish the two clusters along a gradient defined by endemic (cluster C) and introduced (cluster D) forest. Cluster D includes 22 endemic species that are associated with introduced habitat to some extent. In summary, the RDA results showed that a substantial proportion of introduced fly species were associated with introduced vegetation, primarily fruit trees. Endemic fly species were either strongly or completely associated with endemic habitats, or possibly forest habitats that include native plant species, but some of them were able to persist in disturbed habitats, possibly on isolated islands of host plants.

## Discussion

Our results demonstrate the importance of standardized surveys and intensive taxonomy with identifications to species level in contributing to the development of useful measures of habitat quality. Not all endemic species are dependent on high quality native habitat, and a few are largely independent of it. Thus, while most endemic species are more strongly associated with native habitat, relatively few are strongly enough associated to be of the highest value in conservation assessments and used for monitoring environmental health. Such species may be selected among those in clusters C of both Figures 10 and 11. In addition to being restricted to native habitat, ideal attributes of these species or clades should be that they are commonly encountered, easily attracted to bait, diverse in species, and with their taxonomy and host plant associations well documented. These most sensitive species, which could indicate highest quality native habitat and serve as indicators of environmental health should be selected among the *Antopocerus*, spoon tarsus and split tarsus clades. Some of the picture wing species may be considered, because they may be observed and identified in the field without killing them, but several of them do persist in areas distant from endemic forest. While umbrella species *per se* are controversial [30], [31], the use of a broad suite of species, culled from habitat gradient surveys, as employed in this study, can help generate a viable and more nuanced measure of ecosystem integrity less vulnerable to the vagaries of more species-specific management practices.

Conversely, while most alien species surveyed were more strongly associated with disturbed habitats, many were also present or abundant in the highest quality native forest. Thus, a species-level diagnosis of trap composition was needed, not just a total of invasive species, or abundance to understand how invasive flies interact with native habitats. Our species-level approach reveals that some invasive species are useful in assessing habitat integrity, while others invade broadly and probably should be discounted from habitat quality assessments.

Many endemic drosophilids are still relatively common and fairly diverse in endemic habitats, due in part to significant efforts to conserve endemic plants and their associated insect faunas by excluding pigs and other ungulates in fenced forest reserves [18], (e.g. the Ola’a and the Waikamoi Forests). Although we collected 41.5% of the expected species, additional described species may persist, at least as small isolated populations. The species accumulation curves in native forest (Fig. 5a–b) might have approached asymptotes had our sampling been more targeted and less randomized, with active visual searches for adults attracted to bait applied to trees, sampling of larval substrates, and strategically placing our traps near host trees. However, our goal was not to locate particular species, but rather to examine the potential of standardized trapping gradients to identify informative species for conservation management. Future surveys specifically targeting assessments of native habitat integrity should use these more directed techniques to detect the most sensitive species we identified in this study.

The presence of endemic species in mixed alien forest is attributable to dispersal from endemic forest, but probably also persistence on isolated endemic host trees and adaptation to introduced hosts. Heed [10] repeatedly reared nine species from leaf litter of a single isolated *Cheirodendron* tree in Kīpuka Puaulu (Volcanoes N.P.), and *D. reynoldsiae* Hardy and Kaneshiro was reared from the bark of four endemic *Reynoldsia* trees isolated in nonnative forest at Kulu’ī Gulch in the Ko’olau mountains [11]. Thus, the importance of preserving even remnant populations of native plants cannot be underestimated in maintaining endemic Hawaiian drosophilid diversity. Even single trees may serve as ‘island’ refugia from which restoration efforts can be initiated [10]. Thirteen endemic species were reared from plant genera that are not native to Hawai’i, and the majority of these are oligophagous or polyphagous [13]. With intensive sampling, even some of the apparently more sensitive picture wings may be collected at very low altitudes, such as *D. ochracea* in our study and the polyphagous *D. crucigera* Grimshaw, reared from nonnative *Erythrina* bark at 120 m altitude at the Lyon Arboretum (O’ahu) [11], and still present at that site in 2010 (L.L. unpublished data). The ubiquity of some invasive Drosophilidae across even the most intact native forests in our study suggests that these alien species can’t serve as indicators of lower habitat quality, emphasizing the need for species level identifications, which allow the use of more restricted, and informative, species.

The technology used to manage pest fruit flies, although with some undesirable nontarget effects, is a major improvement over the past practice of organophosphorous insecticide cover sprays, and has been widely adopted by Hawaiian growers in recent years [32]. Our study suggests that a few precautions can help conserve endemic drosophilids [21]. Food lure traps and protein bait spraying should be restricted to within orchards and avoided in the vicinity of isolated endemic host plants that may sustain small breeding populations of endemic drosophilids. The deployment of improved male lure formulations that do not require traps [32] should be encouraged for fruit fly control through male annihilation. If these relatively simple measures are taken, the potential nontarget impacts of fly control on native insects can be minimized.

Endemic Drosophilidae represent the most species diverse lineage in Hawai’i and a globally unique resource for understanding the process of evolution [33]. Our research demonstrates that most endemic fly species are restricted to native forest, and that a subset of species is highly sensitive and may be useful indicator species. Standardized gradient trapping schemes like those employed here are also important in generating baseline data for long term habitat monitoring. Over time, the retreat and extinction of particular species, even from forest reserves, may provide important indications of subtle changes in ecosystem integrity that are not otherwise obvious [34]. Interestingly, there is a subset of endemic species that are able to persist in invaded forest, presumably where remnant native host trees occur in very low densities. This is encouraging for conservation efforts since it suggests that even highly degraded indigenous forest areas may maintain, in the short term at least, some of their endemic plant and insect components. Thus conservation efforts in areas that appear to be almost completely overwhelmed by invasive plants may not be a lost cause.

## Supporting Information

Table S1 **Complete List of Species Collected.**
(PDF)
