# Supplementary material for: Conservation Implications of Changes in Endemic Hawaiian Drosophilidae Diversity across Land Use Gradients
Source: PLoS One. 2013 May 1;8(5):e62464. doi: 10.1371/journal.pone.0062464 (PMC3641069; doi:10.1371/journal.pone.0062464)
Supplement: Table S1 — Complete List of Species Collected. (PDF) [file pone.0062464.s001.pdf]

Table S1: Complete List of Species Collected

The following list is a compilation of every species of Drosophilidae collected during our study, with details on the number of sites in which each species was collected within each transect, and the cluster position of each species in Figures 10 and 11 in the main paper.

Taxonomic groupings and species	Hawai‘i							Maui			
	% of total endemics (Hawai‘i)	Stainback # endemic sites (of 4)	Stainback # non-endemic sites (of 5)	Saddle # sites (of 15)	Kohala # sites (of 6)	Waimea # sites (of 5)	Group on Figure 10	% of total endemics (Maui forest)	Maui Forest # sites (of 38)	Kula orchard sites # sites (of 9)	Group on Figure 11
***Drosophila* (*Antopocerus*)**											
D. adunca Hardy, 1965								2.31	28		C
D. cognata Grimshaw, 1901	1.71	4		7	3		B				
D. diamphidopoda (Hardy, 1965)								3.63	36		C
D. orthoptera (Hardy, 1965)								0.12	8		C
D. tanythrix (Hardy, 1965)	37.16	4		9	6		C				
D. villosa (Hardy, 1965)								0.01	1		C
D. yooni Hardy, 1977	0.34			4			C				
***Drosophila* (*Bristle tarsus*)**											
D. brunneisetae Hardy, 1965	0.01	1[b]					C	0			
D. expansa Hardy, 1965	0							0.01	1		C
D. petalopeza Hardy, 1965								0.04	3		C
D. trichaetosa Hardy, 1965	0.05	0[a]		2	0[a]		C				
***Drosophila* (*Ciliate tarsus*)**											
D. imparisetae Hardy, 1965	0.01	0[a]		1	0[a]		C				
D. latigena Hardy, 1965	0	0[a]		0[a]	0[a]			0.06	4		C
D. medialis Hardy, 1966	0.18	0[a]		4	1		C				
D. nigritarsus Hardy, 1965	0.01	0[a]		1			C				
***Drosophila* (*Split tarsus*)**											
D. ancyla Hardy, 1965								0.37	20		C
D. cnecopleura Hardy, 1965	4.70	3		4	6	1	C				
D. cracens Hardy, 1965	0.00							0.01	1		C
D. fundita Hardy, 1965								0.06	6		C
D. paracracens Hardy & Kaneshiro, 1979	0.30			3	2		C				
D. paucitarsus Hardy & Kaneshiro, 1979								0.08	10[c]		D
D. propriofacies Hardy, 1965	0.71			3	0[a]		C	0.00			
D. variabilis Hardy, 1965								0.48	19		C

Taxonomic groupings and species	Hawai‘i							Maui			
	% of total endemics (Hawai‘i)	Stainback # endemic sites (of 4)	Stainback # non-endemic sites (of 5)	Saddle # sites (of 15)	Kohala # sites (of 6)	Waimea # sites (of 5)	Group on Figure 10	% of total endemics (Maui forest)	Maui Forest # sites (of 38)	Kula orchard sites # sites (of 9)	Group on Figure 11
***Drosophila* (*Split tarsus*)**											
D. ancyla Hardy, 1965								0.37	20		C
D. cnecopleura Hardy, 1965	4.70	3		4	6	1	C				
D. cracens Hardy, 1965	0.00							0.01	1		C
D. fundita Hardy, 1965								0.06	6		C
D. paracracens Hardy & Kaneshiro, 1979	0.30			3	2		C				
D. paucitarsus Hardy & Kaneshiro, 1979								0.08	10[c]		D
D. propriofacies Hardy, 1965	0.71			3	0[a]		C	0.00			
D. variabilis Hardy, 1965								0.48	19		C
***Drosophila* (*Spoon tarsus*)**											
D. conformis Hardy, 1965	1.86	3		2	0[a]		C				
D. contorta Hardy, 1965								0.01	1		C
D. dasycnemia Hardy, 1965	2.78	4		3	5		C				
D. incognita Hardy, 1965	0.01	1					C				
D. mimiconformis Hardy, 1965								0.01	2		C
D. neutralis Hardy, 1965	6.78	4		3	6		C				
D. percnosoma Hardy, 1965	8.67	4		6	6		C				
D. sordidapex Grimshaw, 1901	0.84	4		4	3		C				
D. waddingtoni Basden, 1976	0.09	0[a]		2	0[a]		C	10.27	28		C
***Drosophila* (*Picture wing*)**											
D. adiastola Hardy, 1965								0.08	5		C
D. clavisetae (Hardy, 1966)								0.01	2		C
D. cyrtoloma Hardy, 1969								0.06	4		C
D. discreta Hardy & Kaneshiro, 1968								0.11	12		D
D. disjuncta Hardy, 1965								0.06	5		C
D. fasciculisetae Hardy, 1965								1.54	27		D
D. grimshawi Oldenberg, 1914								0.16	16		D
D. lanaiensis Grimshaw, 1901								0.01	1		D
D. limitata Hardy & Kaneshiro, 1968								0.24	11		D
D. melanocephala (Hardy, 1966)								0.03	2		C
D. murphyi Hardy & Kaneshiro, 1969	0.11	1	1	1	3		C				
D. neopicta Hardy & Kaneshiro, 1968								0.01	1		C
D. obscuripes (Grimshaw, 1901)								0.01	1		C
D. ochracea Grimshaw, 1901	1.08	4	4	0[a]	0[a]		C				
D. orphnopeza Hardy & Kaneshiro, 1968								0.12	9		D
D. pilatisetae Hardy & Kaneshiro, 1968								0.03	4		D
D. planitibia (Hardy, 1966)								0.19	11		D
D. recticilia Hardy & Kaneshiro, 1968								0.17	11		C
D. silvestris (Perkins, 1910)	0.13			3			C				
D. sproati Hardy & Kaneshiro, 1968	0.34	2		1	5		C				

	Hawai‘i							Maui			
Taxonomic groupings and species	% of total endemics (Hawai‘i)	Stainback # endemic sites (of 4)	Stainback # non-endemic sites (of 5)	Saddle # sites (of 15)	Kohala # sites (of 6)	Waimea # sites (of 5)	Group on Figure 10	% of total endemics (Maui forest)	Maui Forest # sites (of 38)	Kula orchard sites # sites (of 9)	Group on Figure 11
***Drosophila* (*haleakalae*)**											
D. bipolita Hardy, 1965	3.04	4	2	4	2	1	C	3.36	21		C
D. brunneicrus Hardy & Kaneshiro, 2001	0.04			1			C				
D. canipolita Hardy, 1965	0.12	0[a]		2			C	1.86	33		D
D. cilifemorata Hardy, 1965	0.05	0[a]		1	0[a]		C	0.00	0[a]		
D. demipolita Hardy, 1965	0.20			4			C				
D. dolichotarsis Hardy, 1966								0.23	14		C
D. fungiperda Hardy, 1965	0.16	0[a]		4			C				
D. fuscifrons Hardy, 1965								0.20	9	2	D
D. iki Bryan, 1934	0.00							1.72	33		C
D. illusipolita Hardy, 1965	0.24	4			0[a]		C				
D. inciliata Hardy & Kaneshiro, 1968								0.20	16		C
D. longiperda Kambysellis, 1993	0.17			5	0[a]		C				
D. macrochaetae Hardy, 1965	0.28			4[b]			C	0.00	0[a]		
D. melanoloma Hardy, 1965								0.04	3		C
D. multiciliata Hardy & Kaneshiro, 2001	1.05	1		6			C				
D. nigella Hardy, 1965								23.24	37		D
D. nigra Grimshaw, 1901								2.15	29		C
D. ochropleura Hardy & Kaneshiro, 2001	0.36			4			C				
D. polita Grimshaw, 1901	0.00				0[a]			0.03	3	2	D
D. quinqueramosa Hardy & Kaneshiro, 2001								0.04	4	2	D
D. stenoptera Hardy, 1965								0.01	1		C
***Drosophila* (*Modified mouthparts*)**											
D. comatifemora Hardy, 1965								6.15	30		D
D. desallei Magnacca & O'Grady, 2009	0.11			3	3		C				
D. furva Hardy, 1965								0.01	1		C
D. hirtitarsus Hardy, 1965								0.71	26	1	D
D. infuscata Grimshaw, 1901	0.24	2	2	0[a]	3		C				
D. mitchelli Hardy, 1965	0.03	0[a]		0[a]	1		C				
D. nigrocirrus Hardy, 1965	0.08	0[a]		2	0[a]		C				
***Drosophila* (*Nudidrosophila*)**											
D. makawao Magnacca & O'Grady, 2008								0.67	21		D
D. pohaka Magnacca & O'Grady, 2008	0.46	3		3			C	0.00			

Taxonomic groupings and species	Hawaiʻi							Maui			
	% of total endemics (Hawaiʻi)	Stainback # endemic sites (of 4)	Stainback # non-endemic sites (of 5)	Saddle # sites (of 15)	Kohala # sites (of 6)	Waimea # sites (of 5)	Group on Figure 10	% of total endemics (Maui forest)	Maui Forest # sites (of 38)	Kula orchard sites # sites (of 9)	Group on Figure 11
***Drosophila* (*unplaced*)**											
D. joycei Hardy, 1965								0.06	5		C
***Scaptomyza* (*Alloscaptomyza*)**											
S. buccata Hackman, 1962	0.00							0.17	15	1	C
S. mutica Hardy, 1965	0.13			2[b]			D	0.00	0[a]		
***Scaptomyza* (*Bunostoma*)**											
S. bryanti Hackman, 1959	0.00							0.04	4		C
S. confusa Hardy, 1965								n.a.		3	B
S. palmae Hardy, 1965					0[a]	1	D				
S. xanthopleura Hardy, 1965								0.01	2[c]	3[c]	D
***Scaptomyza* (*Elmomyza*)**											
S. affinicuspidata Hardy, 1965								0.05	4		C
S. articulata Hardy, 1965	0.00			0[a]				0.02	2[c]		D
S. basiloba Hardy, 1965	0.00			0[a]				3.23	17[d]		C
S. concinna Hardy, 1965								0.04	3		C
S. cryptoloba Hardy, 1965	8.76	4[b]	2[b]	9[b]	6[b]		C	0.00			
S. cuspidata Hardy, 1965	0.00	0[a]						0.08	7		C
S. cyrtandrae Hardy, 1965	0.22	1		1			C				
S. decepta Hardy, 1965								7.13	30	2	C
S. diaphorocerca Hardy, 1965								0.04	4[c]	1[c]	C
S. domita Hardy, 1965								0.01	1		D
S. exigua (Grimshaw, 1901)	9.14			11	2		C	0.00	0[a]		
S. hackmani Hardy, 1965	0.09			3	0[a]		C	0.01	1	1	C
S. inaequalis (Grimshaw, 1901)	4.74	0[a]		7			D	0.00	0[a]		
S. intricata Hardy, 1965								1.55	24		C
S. levata Hardy, 1965	0.00				0[a]			n.a.		1[c]	B
S. lobifera Hardy, 1965	0.01				1	3	D				
S. longipecten griseonigra Hardy, 1965								0.01	1		C
S. ochromata Hardy, 1965								13.14	37		C
S. retusa Hardy, 1965								0.01	1		C
S. silvicola Hardy, 1965	0.00							0.64	19		C
S. tumidula Hardy, 1965	0.67			9			C				
S. umbrosa Hardy, 1965	0.00							0.01	2		C

Taxonomic groupings and species	Hawai‘i							Maui			
	% of total endemics (Hawai‘i)	Stainback # endemic sites (of 4)	Stainback # non-endemic sites (of 5)	Saddle # sites (of 15)	Kohala # sites (of 6)	Waimea # sites (of 5)	Group on Figure 10	% of total endemics (Maui forest)	Maui Forest # sites (of 38)	Kula orchard sites # sites (of 9)	Group on Figure 11
***Scaptomyza* (*Engiscaptomyza*)**											
S. crassifemur (Grimshaw, 1901)								12.77	35	1	C
S. nasalis (Grimshaw, 1901)								0.04	3	1	C
***Scaptomyza* (*Exalloscaptomyza*)**											
S. mauiensis (Grimshaw, 1901)								0.01	1	2	B
***Scaptomyza* (*Grimshawomyia*)**											
S. undulata (Grimshaw, 1901)	1.38	1		7			C				
***Scaptomyza* (*Tantalia*)**											
S. brunnimaculata Hardy, 1965								0.01	1[c]		C
S. nigrosignata Hardy, 1965	0.00							0.01	1[c]		D
***Scaptomyza* (*Titanochaeta*)**											
S. bryani (Wirth, 1952)	0.00				0[a]			0.03	4		C
S. chauliodon (Hardy, 1965)	0.01			1[b]		1[b]	C	0.01	1		D
S. setosiscutellum (Hardy, 1965)	0.00				0[a]			0.04	5[c]		C
***Scaptomyza* (*Unplaced*)**											
S. parva (Grimshaw, 1901)	0.30	4		4	4		C				

Taxonomic groupings and species	Hawaiʻi							Maui			
	% of total endemics (Hawaiʻi)	Stainback # endemic sites (of 4)	Stainback # non-endemic sites (of 5)	Saddle # sites (of 15)	Kohala # sites (of 6)	Waimea # sites (of 5)	Group on Figure 10	% of total endemics (Maui forest)	Maui Forest # sites (of 38)	Kula orchard sites # sites (of 9)	Group on Figure 11
**Introduced Drosophilidae**											
Cacoxenus perspicax (Knab, 1914)										3	B
Chymomyza procnemis (Williston, 1896)									1	7	B
Dettopsomyia nigrovittata (Malloch, 1924)					1	1	C				
D. ananassae Doleschall, 1858 and/or *D. kikkawai* Burla, 1954			4	1	1		A				
D. bizonata Kikkawa & Peng, 1938					3	5	D		8	7	A
D. busckii Coquillett, 1901			2		5	3	B		31	9	D
D. cardini Sturtevant, 1916						1	D			3	B
D. hydei Sturtevant, 1921							D				B
D. immigrans Sturtevant, 1921		4	5	8	6	4	A		19	8	D
D. lutzii Sturtevant, 1916				3	2	3	D		12	7	B
D. melanogaster Meigen, 1830		1	2			1				4	B
D. quadrilineata deMejeire, 1911		2	3		5	2			4	1	D
D. repleta Wollaston, 1858 and/or *D. mercartorum* Patterson & Wheeler, 1942										5	B
D. simulans Sturtevant, 1919		1	1		1	3	C			6	B
D. sulfurigaster bilimbata Bezzi, 1928		3	5	6	2	4	A		1	1	C
D. suzukii (Matsumara, 1931)		4	5	15	6	5	B		37	8	D
Hirtodrosophila sp. nr. *unicolorata* Wheeler, 1959				4	3	2	C		2	3	B
Leucophenga maculosa (Coquillett, 1895)			1	1	1	3	C		2	9	B
Scaptodrosophila sp			2				D				
Scaptomyza elmoi Takada, 1970				1	5	3	B		8	5	C
Zaprionus ghesquierei Collart, 1937			2				D		1	6	B

0[a] Known to be present in sampled area but not collected during our surveys
[b] New island record for Hawaiʻi Island
[c] New island record for Maui Island
[d] *S. basiloba* is a new island record for Maui Island, or it may be an undescribed closely related species
